# Probiotics in Term Infants: Clinical Impact of Infant-Type Bifidobacteria: A Systematic Review and Meta-analyses

**DOI:** 10.1016/j.tjnut.2025.10.006

**Published:** 2025-10-09

**Authors:** Mathias A Sjælland, Marie T Philipsen, Tine B Henriksen, Josephine Skipper, Sune Rubak

**Affiliations:** 1 Department of Clinical Medicine, Aarhus University, Aarhus, Denmark; 2 Department of Paediatrics and Adolescent Medicine, Aarhus University Hospital, Aarhus, Denmark

**Keywords:** probiotics, Bifidobacteria, child health, immune system, gut microbiota

## Abstract

Probiotics labeled "infant-type bifidobacteria" (ITB), such as the *B. longum* subsp. *infantis* strains, have gained significant attention in recent years for their potential to positively influence the gut microbiome, early immune system development, and consequently future health. However, significant knowledge gaps remain regarding the actual clinical impact, the optimal strains, dosing regimens, and treatment duration—both in general and for ITB-probiotics specifically. This systematic review evaluates the clinical effects of administering ITB-probiotics to healthy, term infants within the first year of life. The aim was to address all categories of clinical outcomes. However, the included studies focused primarily on antibiotic use, atopic conditions, gastrointestinal health, and growth. We systematically and comprehensively searched PubMed, Embase, CENTRAL, and Scopus, followed by a meta-analysis where applicable. A total of 25 studies were included and assessed for risk of bias using the revised Cochrane tool (RoB 2). We found that early administration of ITB-probiotics was associated with a significant reduction in eczema [risk ratio (RR) = 0.78 (0.68, 0.90)] and a borderline significant reduction in respiratory tract infections [RR = 0.74 (0.54, 1.00)]. Other commonly reported outcomes, including antibiotic use, diarrhea, asthmatic bronchitis, and food allergy, also showed trends toward a preventive effect, though these findings did not reach statistical significance. This review underscores the potential clinical relevance of ITB-probiotics, particularly in the prevention of eczema and respiratory tract infections. However, the evidence is limited by study heterogeneity and a lack of long-term follow-up data. Further high-quality randomized controlled trials with larger sample sizes and standardized outcome measures are needed to clarify both short- and long-term effects of ITB probiotic administration in neonates and infants.

This trial was registered at PROSPERO as CRD42024507608**.**

## Introduction

The International Scientific Association for Probiotics and Prebiotics defines probiotics as “live microorganisms that, when administered in adequate amounts, confer a health benefit on the host” [1].

However, it remains unclear whether many probiotics actually provide their claimed benefits. Despite this uncertainty, the use of probiotics, with various bacterial strains, has rapidly increased among the general population. This highlights an important knowledge gap regarding the evidence of specific probiotic mechanisms and their potential in a clinical setting, both preventively and as a treatment [2].

*Bifidobacterium* is the predominant genus in the infant gut microbiome, but over the past decades, there has been a generational decline in the prevalence [3]. This lack of bifidobacteria has been associated with systemic inflammation and immune imbalance early in life [4]. *Bifidobacterium longum* subsp*. infantis (B. infantis)* is especially well-suited to efficiently utilize human milk oligosaccharides (HMOs) as a carbon and energy source, creating short-chain fatty acids that are key components in pathways driving early infant growth and immune system development. *B. bifidum* and, to a lesser extent, *B. breve* and *B. longum* subsp. *longum,* also have similar properties [5]. Thereto, recent studies demonstrated the ability of these bacteria to convert aromatic amino acids into aromatic lactic acids, which is also believed to influence the development of the human immune system [6]. Because of their predominance in early childhood, these will be referred to as “infant-type”-bifidobacteria (ITB). These qualities and their Generally Recognized As Safe status have made bifidobacteria a commercially popular probiotic agent despite only little high-quality scientific evidence [7,8]. Nevertheless, a growing body of research on gut microbiota underscores the potential role in addressing various pediatric health concerns. Studies have associated the increasing prevalence of pediatric allergic disorders with the gradual alteration in the gastrointestinal microbiota of infants [9]. Previous reviews have demonstrated a preventive effect of some probiotics on the development of eczema [10,11]. These findings align with research suggesting that changes in the infant gut microbiome modulate the immune system, potentially influencing not only allergic and atopic diseases but also infections, inflammatory conditions, and autoimmune disorders in general [12].

Previous reviews within this area either: *1)* considered “probiotics” as a single entity, with no (or limited) strain differentiation *2)* evaluated probiotics solely as a treatment, rather than assessing the preventive potential effect in healthy infants; or *3)* focused solely on short-term outcomes.

According to The World Gastroenterology Organisation, recommendations of probiotics in a clinical setting should be both strain specific, tied to the unique properties of the strain, and based on human trials [13]. This calls for improved future trials and focused systematic reviews evaluating the effects of probiotics.

Therefore, this review specifically aims to evaluate the preventive effects of probiotics with ITB by assessing studies with clinical outcomes after administration to healthy, term infants. Gut colonization, inflammatory markers, immune cell activity, and other biochemical markers are not included in this review.

## Methods

This systematic review was performed in accordance with the PRISMA 2020 statement [14].

### Search strategy

PubMed, Embase, CENTRAL, and Scopus were searched on 13 February, 2024, without any restriction on language or date of publication. The search was updated on 29 November, 2024.

The search string (Text S1) was developed with a liaison librarian at Aarhus University.

### Eligibility criteria

The inclusion criteria were based on the Population, Intervention, Comparison, and Outcomes (PICO) framework [15].

Studies met the inclusion criteria if they included: *1)* a population of human infants born at 37 gestational weeks or more and <1 y of age at study start; *2)* oral administration of probiotics containing any of the ITB (*B. longum* subsp. *infantis, B. bifidum, B. breve,* and *B. longum* subsp. *longum*), given alone or in combination with each other or other probiotics or synbiotics; *3)* a comparator group receiving either placebo or no probiotic supplement; and *4)* ≥1 reported clinical outcome.

Both randomized controlled trials (RCTs) and observational studies, including cohort and case-control studies, were considered eligible for inclusion.

Studies were excluded if they were case studies or case reports. Studies performed in a patient population were also excluded to avoid evaluating probiotics as treatment for any pre-existing condition.

Two reviewers (JS and MAS) independently screened the studies using Covidence [16] and reached consensus upon discussion. The first step included title and abstract screening, and the second step was full-text screening. Disagreements between the 2 independent reviewers were resolved by a third senior collaborator (SR or TBH).

### Quality assessment and data extraction

The risk-of-bias assessment for randomized trials included randomization, deviation from intended treatment, missing outcome data, measurement of the outcome, and selection of the reported result using the revised Cochrane tool (RoB 2) [17].

The application of the risk-of-bias tool was performed by 2 authors working independently (JS and MAS). The entire review team of 5 people (JS, MAS, MTP, TBH, and SR) was involved in the discussion of the quality assessment.

A full RoB 2 assessment was conducted for outcomes summarized with a meta-analysis of results. If a study with a high risk of bias significantly influenced the results of the meta-analysis, this was explicitly stated in the text.

The quality assessment is visualized using the RoBvis tool as weighted bar plots of the distribution of risk-of-bias judgments within each bias domain.

Data extraction was performed by 2 reviewers (JS and MAS) independently using standardized templates created within Covidence.

### Statistical analyses

Stata 18.0 (StataCorp) was used to perform the statistical analyses and the visual display of the meta-analyses. When ≥3 studies used a comparable outcome measure, a meta-analysis was conducted. Continuous outcomes were expressed as the standardized mean difference (MD) or the MD, with the difference calculated as the control/placebo group mean minus the intervention group mean with a 95% confidence interval (CI). Dichotomous outcomes were presented as risk ratios (RRs) with a 95% CI, where the ratio represented the intervention group risk divided by the control (placebo) group risk. The random effects restricted maximum likelihood (REML) model was used for all outcomes. Statistical heterogeneity was assessed using the *I*^2^ statistic and was interpreted according to the Cochrane Handbook recommendations [18].

To assess potential publication bias, funnel plots were generated and Egger’s regression test for small-study effects was performed when >10 studies reported on the same outcome.

For some outcomes, it was necessary to calculate an SD from the IQR or the SE. When results were presented as a median and IQR, the sample mean and SD were estimated and evaluated for skewness to enable their application in the meta-analysis [19,20].

Unless otherwise indicated by the study authors, loss to follow-up was assumed to be random. No further imputations were made for missing data. In cases of unclear or missing outcome data, study authors were contacted for clarification when possible.

A leave-one-out sensitivity analysis was conducted for outcomes with >5 studies, where a single study contributed over 40% of the statistical weight.

As shown in Table 1 [[21], [22], [23], [24], [25], [26], [27], [28], [29], [30], [31], [32], [33], [34], [35], [36], [37], [38], [39], [40], [41], [42], [43], [44], [45], [46], [47], [48], [49], [50], [51], [52], [53]], some studies had >1 intervention fitting the inclusion criteria for this review; these groups were combined. Subgroup analyses were performed when >10 studies evaluated the outcome. The subgroup analyses were based on the primary areas of variance among the included studies as defined by the PICO framework. Some of these are visualized in Table 1.

**TABLE 1:** Characteristics of the 25 studies evaluating ≥1 clinical outcome after administration of ITB (infant-type Bifidobacteria) probiotics

Author (references), year	Intervention(s) and control (*+n*)	Age at study start (d)	Duration (wk)	Feeding pattern	Risk status
Abrahamse-Berkeveld et al. [21], 2016	Synbiotic: (*n =* 105) Formula + *B. breve* M16V + scGOS +lcFOS Placebo *(n = 123)*	<35	13	Formula	÷
Alba et al. [22], 2024	Formula + *B. breve* DSM32583 (*n =* 90) Placebo (formula) (*n =* 97)	90	12	Formula	÷
Allen et al. [23,24], 2014	Multistrain probiotic: (*n = 220*) *L. salivarius* CUL6 + *L. paracasei* CUL08 + *B. animalis* subsp. *lactis* CUL3 + *B. bifidum* CUL20 Placebo (*n* = 234)	4 w PN and from 0	26	Both	+
Bellomo et al. [25], 2024	*B. bifidum* PRL2010 (*n* = 164) No intervention (*n* = 249)	3	52	Both	C-sec
Capeding et al. [26], 2023	*B. longum* subsp*. infantis* LMG11588 (low-dose and high-dose group) (*n* = 151) Placebo (*n* = 77)	14–21	8	Both	÷
Chouraqui et al. [27], 2008	Synbiotic: (*n* = 70) *B. longum* BL999, *L. rhamnosus* LPR, and GOS/SCFOS Synbiotic: (*n* = 74) *B. longum BL999*, *L. paracasei* ST11, and GOS/SCFOS Multistrain probiotic: (*n* = 70) *B. longum* BL999 and *L. rhamnosus* LPR Placebo (formula) (*n* = 70)	<14	14–16	Formula	÷
Dissanayake et al. [28], 2019	Synbiotic: (*n* = 137) *B. bifidum* OLB6378 + FOS No intervention (*n* = 137)	0	26	Both	÷
Enomoto et al. [29], 2014	Multistrain probiotic: (*n* = 130) *B. longum* BB536 and *B. breve* M-16V No intervention (*n* = 36)	4 w PN and from 7	26	Both	÷
Escribano et al. [30], 2019	Formula + *B. longum* subsp. *infantis* CECT7210 (*n* = 93) Placebo (formula) (*n* = 97)	<120	12	Formula	÷
Giglione et al. [31,32], 2016	*B. breve* B632 and BR03 (*n* = 29) Placebo (*n* = 31)	<15	12	Both	÷
Harvey et al. [33], 2014	Synbiotic: (*n* = 59) Formula + *B. breve* M-16V + FOS Placebo (formula) (*n* = 56)	<15	16	Formula	÷
Hascoët et al. [34], 2011	Formula + *B. longum* BL999 (*n* = 40) Placebo (formula) (*n* = 39)	<7	16	Formula	÷
Hiraku et al. [35], 2023	*B. longum* subsp. *infantis* M-63 (*n* = 57) Placebo (*n* = 54)	<7	12	Both	÷
Hoy-Schulz et al. [36], 2016	Multistrain probiotic: (*n* = 40) *L. reuteri* and *B. longum* subsp. *infantis* 35624 No intervention (*n* = 40)	28–120	4	Both	÷
Kukkonen et al. [[37], [38], [39], [40], [41]], 2007	Synbiotic: (*n* = 610) *L. rhamnosus* GG; *L. rhamnosus* LC705, *B. breve* Bb99 and *P. freudenreichii* subsp. *shermanii* JS + sugar syrup and GOS Placebo (*n* = 613)	2–4 w PN and from 0	26	Both	+
Maldonado et al. [42], 2019	Formula + *B. breve* CECT7263 (*n* = 76) Placebo (formula) (*n* = 77)	30	48	Formula	÷
Manzano et al. [43], 2017	*B. longum*. subsp. *infantis* R0033 (*n* = 53) *B. bifidum* R0071 (*n* = 51) Placebo (*n* = 52)	90–365	8	Both	÷
Niers et al. [44,45], 2009	Multistrain probiotic: (*n* = 78) *B. bifidum* W23, *B. lactis* W52 and *Lc. lactis* W58 Placebo (*n* = 78)	8 w PN and from 0	52	Both	+
Phavichitr et al. [46], 2021	Synbiotic: (*n* = 163) scGOS/lcFOS and *B. breve* M-16V (low-dose and high-dose group) Placebo (*n* = 84)	43–65	6	Formula	÷
Puccio et al. [47], 2007	Formula + *B. longum* BL999 (*n* = 69) Placebo (formula) (*n* = 69)	<14	16	Formula	÷
Rozé et al. [48], 2012	Synbiotic: (*n* = 48) *L. rhamnosus* LCS742 and *B. longum* subsp. *infantis* M63 + GOS and scFOS and bovine a-lactalbumin Placebo (formula) (*n* = 49)	<3	26	Formula	÷
Smilowitz et al. [49,50], 2017	*B. longum* subsp. *infantis* EVC001 (*n* = 41) Lactation support (*n* = 39)	7	3	Breastfed	÷
Soh et al. [51], 2009	Multistrain probiotic: (*n* = 127) *B. longum* BL999 and *L. rhamnosus* LPR + formula Placebo (formula) (*n* = 126)	0	26	Formula	+
Wang et al. [52], 2021	Synbiotic: (*n* = 112) Formula + scGOS/lcFOS (9:1) + *B. breve* M-16V Formula (placebo) (*n* = 112)	<44	12	Formula	÷
Xiao et al. [53], 2019	Multistrain probiotic: (*n* = 55) *B. longum* subsp*. infantis* R0033, *B. bifidum* R0071, and *L. helveticus* R0052 Placebo (*n* = 59)	105–180	4	Formula	÷

The included studies were all randomized controlled trials. Interventions are classified as single-strain probiotics (no title), “multistrain probiotic,” or “symbiotic.” ITB strains are highlighted. Risk status refers to a deliberate link between inclusion criteria and 1 or more study outcomes.

Abbreviations: B, bifidobacterium; GA, gestational age; GOS, galactooligosaccharides; PN, prenatal; sc(lc)FOS, short-chain (long-chain) fructooligosaccharides; +, Family history of allergic disease (Allen et al. intended to recruit only “high-risk” infants); ÷, General or unselected infant population; C-sec, Only cesarean-born infants.

## Results

### Study characteristics

Of the 7826 articles screened (title and abstract), 78 were assessed for eligibility by a full-text review, and 31 articles describing a total of 25 individual studies were included (Supplemental Figure 1). Although both observational studies and case-control studies were eligible for inclusion, all studies identified were RCTs. Articles referring to the same study are labeled with the first author of the first article (see Table 1). In all tables and figures, studies are listed alphabetically.

The publication dates range from 2007 to 2024, and the studies were conducted in North America (*n =* 2), Europe (*n =* 14), and Asia (*n =* 9). The interventions included single-strain probiotics (*n =* 11), multistrain probiotics (*n =* 6), synbiotics (*n =* 7), and 1 that involved multiple groups with both syn- and multistrain probiotics. The probiotics used were either ITB alone (*n =* 17) or mixed with ≥1 other type of probiotic (*n =* 8).

The age at the beginning of the intervention varied from 0 days to 4 mo, with 16 studies starting the intervention in infants <1 mo old. Four studies included prenatal administration. The administration periods ranged from 3 to 52 wk, with most studies <6 mo (*n =* 16) and some ≥6 mo (*n =* 9). The infants were either exclusively formula-fed (*n =* 13), exclusively breastfed (*n =* 1), or the study had no restrictions regarding the feeding pattern of the infants (*n =* 11).

Table 1 provides a more detailed description of the individual study characteristics.

Supplemental Table 1 provides an overview of the outcomes in each study.

### Summary of findings from the meta-analyses

**FIGURE 1:**
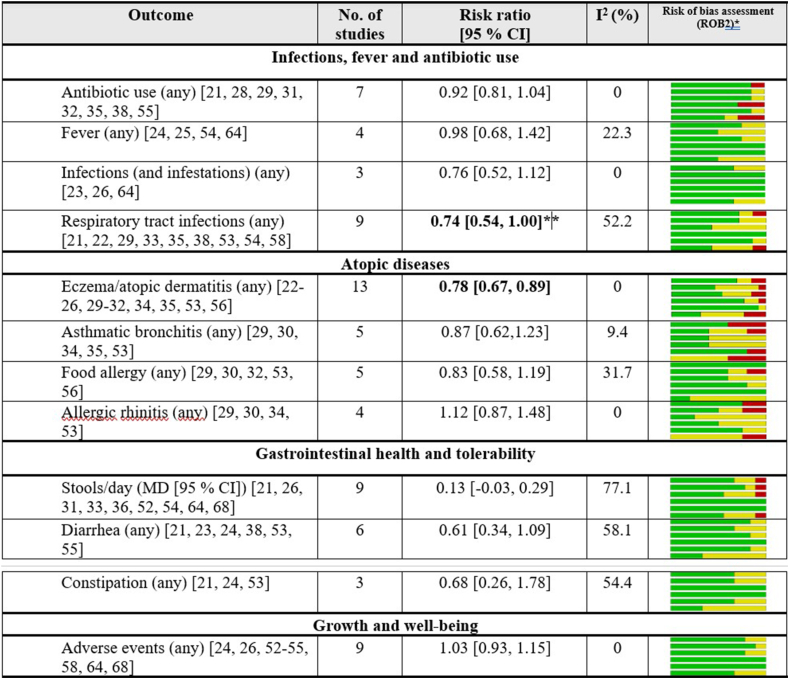
Meta-analyses results [RR (95% CI)] for clinical outcomes assessed in >3 studies evaluating the effect of ITB probiotic administration. Heterogeneity (*I*^2^) and a visual presentation of the risk-of-bias assessment (RoB 2) are included. ∗The bars represent potential bias from: *1*) randomization process, *2*) deviations from intended interventions, *3*) missing outcome data, *4*) measurement of the outcome, *5*) selection of the reported result and *6*) is the overall risk of bias. Green = low risk, yellow = medium risk, and red = high risk. ∗∗Excluding studies with a high risk of bias. CI, confidence interval; ITB, infant-type bifidobacteria; MD, mean difference; RR, risk ratio.

Figure 1 provides an overview of the results of the meta-analyses. Stools/day was the only continuous outcome consistently evaluated in >3 studies. Outcome definitions are provided in later sections. Supplemental Table 2 presents the results of the leave-one-out sensitivity analyses.

### Infections, fever, and antibiotic use

Eight studies examined the use of antibiotics during the follow-up period. Kukkonen et al., Rozé et al. [48], Smilowitz et al. [49], and Soh et al. [51] relied on parental reports, whereas the remaining studies used or appeared to use prescription data.

**FIGURE 2:**
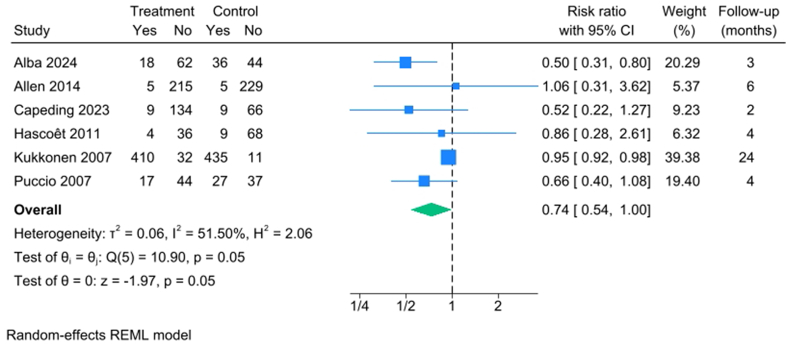
Forest plot of the effect of ITB-probiotics on any antibiotic use in infants during follow-up. Allen et al. [24] began follow-up at 2 mo of age and Kukkonen [37] after 5 y. The remaining studies began follow-up at birth. CI, confidence interval; ITB, infant-type bifidobacteria; REML, restricted maximum likelihood.

The forest plot (Figure 2) [[22], [24], [26], [34], [37], [47]] shows a potential, yet nonsignificant reduction in subsequent antibiotic use among infants given probiotics compared with controls.

Four studies [26,35,[42], [43]] had parental-reported “any fever” as a secondary outcome (Supplemental Figure 2).

Only one of these studies [42] described their definition of fever (>38 °C). The prevalence of fever in this study was close to 50%, whereas Hiraku et al. [35] only reported a prevalence of fever in the cohort of ∼3%. The results did not indicate any effect on the development of fever after ITB-probiotics (Figure 1).

Three studies [[33], [43], [52]] included “Infections and Infestations” as adverse events (AEs), hereby reporting all physician-diagnosed infections (Supplemental Figure 3). The remaining studies reported only selected types of infections and are not included in the meta-analysis. The results suggested a risk reduction similar to that seen for respiratory tract infections (RTIs), though not statistically significant (Figure 1)

Nine studies [[22], [23], [25], [26], [30], [34], [37], [47], [49]] included either “any respiratory infection” or “any upper respiratory infection.” Three studies with a high risk of bias contributed with 21% of the statistical weight and are not presented in the forest plot [25,[30], [49]].

**FIGURE 3:**
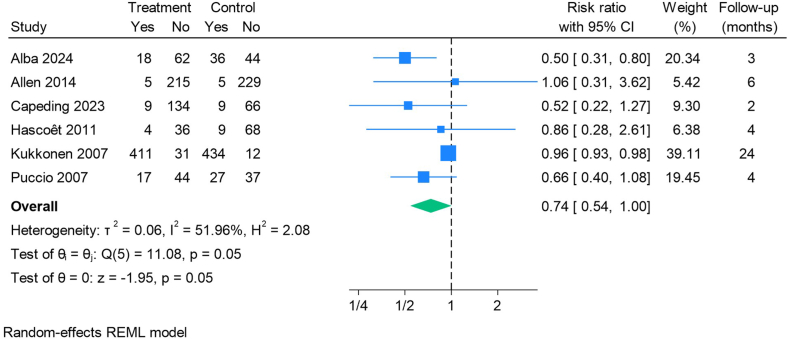
Forest plot of the effect of infant-type bifidobacteria probiotics on the development of any RTI∗ during follow-up. ∗Reports including only "lower respiratory tract infections" have not been included to increase comparability. CI, confidence interval; RTI, respiratory tract infection; REML, restricted maximum likelihood.

The forest plot (Figure 3) shows a trend toward a reduced risk of RTI among children receiving probiotics compared with controls though the result borders statistical significance.

### Atopic diseases

#### Atopic dermatitis

A total of 13 studies included results on the development of eczema/atopic dermatitis. In most of the studies, the terms eczema and atopic dermatitis were used interchangeably. Five studies relied on diary reports on AEs from parents [25,33,35,42,52], whereas the rest of the studies included a clear definition of atopic dermatitis/eczema (the UK Working Party’s diagnostic criteria: *n =* 4, the Japanese Dermatological Association: *n =* 2 and Other: *n =* 3).

The SCORAD ("SCORing Atopic Dermatitis") index [57] was used for severity scoring of atopic dermatitis in 5 [23,37,44,48,51] of these studies. Sufficient data regarding SCORAD were only available from 2 studies [23,44].

**FIGURE 4:**
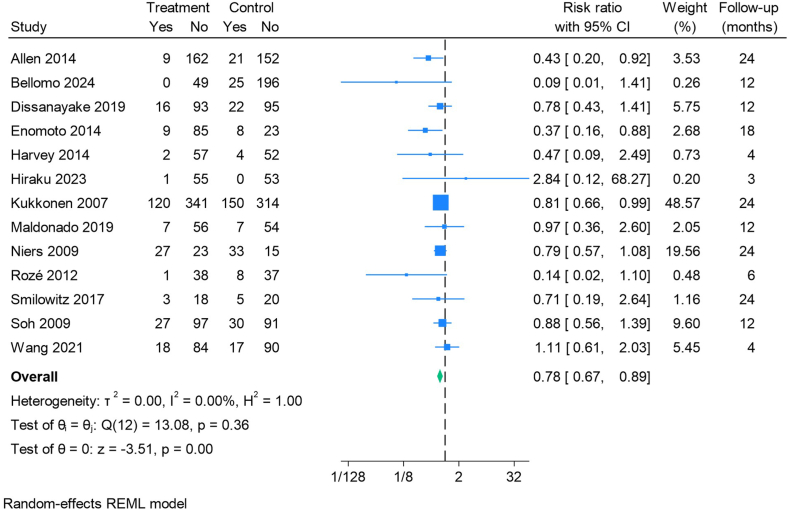
Forest plot of the effect of infant-type bifidobacteria probiotics on the development of eczema/atopic dermatitis during follow-up. CI, confidence interval; REML, restricted maximum likelihood.

The forest plot (Figure 4) shows a statistically significant risk reduction in the development of eczema/atopic dermatitis among the group of infants receiving ITB-probiotics.

The subgroup analyses revealed no significant differences when grouping and comparing studies on PICO differences (Table 1), except for feeding type. Studies without feeding type limitations showed a risk ratio (RR) of 0.75 (0.64, 0.88), compared with an RR of 0.89 (0.64, 1.24) in studies where infants were exclusively formula-fed. Risk reduction remained similar after excluding studies with high risk of bias (RoB) (Supplemental Table 3).

Egger’s regression test for small-study effects showed no significant evidence of publication bias (*P* = 0.11).

#### Other atopic diseases

Some of the studies assessing eczema/atopic dermatitis included other atopic diseases as secondary outcomes, as shown in Table 2 [24,28,29,37,44,49,51].

**TABLE 2:** Elaboration of dichotomous outcomes and results for atopic diseases besides eczema/atopic dermatitis

Study	Outcome measure	Total prevalence	Follow-up period (y)	Risk ratio with 95% CI
Asthma				

Allen et al. [24], 2014	Self-reported asthma	43/350 (12.3%)	0–2	1.20 (0.69, 2.11)
Enomoto et al. [29], 2014	Doctor-diagnosed allergic asthma	3/125 (2.4%)	0–1.5	0.66 (0.06, 7.03)
Kukkonen et al. [37], 2007	Self-reported, doctor-diagnosed asthma	95/642 (14.8%)	0–13	0.75 (0.52, 1.09)
Niers et al. [44], 2009	Current asthma (active in the last 12 mo)	13/83 (15.7%)	0–6	0.71 (0.25, 1.98)
Smilowitz et al. [49], 2017	Self-reported, doctor-diagnosed asthma	1/41 (2.4%)	0–2	3.14 (0.14, 72.92)

Food allergy (FA)				

Allen et al. [24], 2014	Skin prick test positive at either 6 mo or 24 mo for cow milk or eggs	35/344 (10.2%)	0–2	0.40 (0.20, 0.82)
Dissanayake et al. [28], 2019	Self-reported FA	30/223 (13.5%)	0–1	1.06 (0.55, 2.07)
Kukkonen et al. [37], 2007	Self-reported doctor-diagnosed FA	159/642 (24.8%)	0–13	0.84 (0.64, 1.11)
Niers et al. [44], 2009	Doctor-diagnosed FA + sensitization to food allergens	6/83 (7.2%)	0–6	2.26 (0.44, 11.65)
Soh et al. [51], 2009	Any positive dietary skin prick test after 1 y	13/245 (5.3%)	0–2	1.14 (0.39, 3.29)

Allergic rhinitis				

Allen et al. [24], 2014	Self-reported or doctor diagnosed	20/391 (5.1%)	0–2	1.06 (0.45, 2.48)
Enomoto et al. [29], 2014	Doctor diagnosed	0/125 (0%)	0–1.5	0.24 (0.01, 16.63)
Kukkonen et al. [37], 2007	Doctor diagnosed	135/459 (29.4%)	0–13	1.12 (0.89, 1.49)
Niers et al. [44], 2009	Doctor diagnosed—current allergic rhinitis	6/84 (7.1%)	0–6	5.64 (0.69, 46.22)

Abbreviation: CI, confidence interval.

The meta-analyses of these outcomes did not reveal a clear association. However, although the findings suggested a potential risk reduction for asthma and FA following ITB supplementation, they indicated a possible increased risk of allergic rhinitis (Supplemental Figures 4–6). This trend was primarily driven by the Kukkonen et al. [37] trial, which had a larger cohort, a longer follow-up and a higher prevalence of all atopic outcomes compared with the other studies.

### Gastrointestinal health and tolerability

Stool frequency and consistency were the most reported outcomes related to gastrointestinal health and tolerability. Twelve studies reported stool consistency as an outcome, but data were only available in 3 studies. The rest either presented their data in a figure (boxplots, pie charts, etc.) or concluded a significant/nonsignificant difference without presenting the underlying data. Stool frequency was reported as an outcome in 14 studies and data were available from 9 of these.

The overall meta-analysis showed an MD in stools/d of 0.13 (−0.03, 0.29) (Supplemental Figure 7).

Stool frequency was measured at time points between 4 and 26 wk of infant age. A subgroup meta-analysis showed that studies measuring at ≥16 wk (*n =* 3) [[47], [48], [52]] had an MD of 0.27 (0.12, 0.42) (Supplemental Figure 8).

“Any diarrhea” was reported in 6 studies (Supplemental Figure 9) and “any constipation” (Supplemental Figure 10) in 3 studies. Some of these did not include a definition of the outcome measure, as in Table 3 [22,24,27,30,33,35,42]. Alba et al. [22] and Chouraqui et al. [27] reported significant reductions in the number of infants having “any diarrhea episodes” (≥3 loose or watery stools in 24 h). Escribano et al. [30] and Maldonado et al. [42] used the same definition of diarrhea, but did not report a significant association.

**TABLE 3:** Dichotomous outcomes and results for gastrointestinal health

Study	Outcome measure (self-reported)	Total prevalence	Follow-up period	Results
Diarrhea				Risk ratio with 95% CI

Alba et al. [22], 2024	≥3 loose or watery stools in 24 h^1^	18/160 (11.3%)	6 mo	0.29 (0.10, 0.83)
Allen et al. [24], 2014	Undefined	118/386 (30.6%)	6 mo	1.17 (0.86, 1.58)
Chouraqui et al. [27], 2008	≥3 watery stools in 24 h	36/141 (25.5%)	1 y	0.48 (0.28, 0.83)
Escribano et al. [30], 2018	≥3 watery stools in 24 h	10/151 (6.6%)	3 mo	0.46 (0.12, 1.76)
Harvey et al. [33], 2014	Undefined	5/115 (4.3%)	4 mo	1.42 (0.25, 8.20)
Hiraku et al. [35], 2023	Undefined increase in frequency and fluidity of stool compared with baseline	5/109 (4.6%)	3 mo	0.24 (0.03, 2.09)
Maldonado et al. [42], 2019	Loose or watery stool ≥3 times/d	91 events in total	1 y	IRR = 1.13 (0.73, 1.75)^2^

Constipation				Risk ratio with 95% CI

Allen et al. [24], 2014	Undefined	117/386 (30.3%)	6 mo	0.91 (0.67, 1.23)
Escribano et al. [30], 2018	<1 hard stool/d or <1 stool/2 d	12/143 (8.4%)	3 mo	0.19 (0.04, 0.84)
Hiraku et al. [35], 2023	Undefined	5/109 (4.6%)	3 mo	1.42 (0.25, 8.16)

Abbreviation: CI, confidence interval.

1 Outcome defined as “gastrointestinal infection” in article.

2 This study reported number of events and was not included in the meta-analysis.

ITB probiotic use was associated with a potential reduction in risk of both diarrhea and constipation, with the effect on diarrhea approaching statistical significance.

Some studies reported vomiting, but most lacked a distinction between vomiting and regurgitation. The results showed no differences and/or had a very low overall prevalence (Supplemental Table 4).

### Well-being and growth

Most studies did not include a clear definition of colic or used crying time instead. Giglione et al. [32] is the only study not reporting episodes, but total minutes of crying time. It is also the only study reporting a significant effect (Table 4) [21,24,32,35,36,42,49].

**TABLE 4:** Elaboration of outcomes and results concerning crying and colic

Study	Outcome measure	Follow-up period	Result
Abrahamse-Berkeveld et al. [21], 2016	Severity scores: 0 = absent; 1 = mild; 2 = moderate; 3 = severe	Weeks 8–13	Mean = 0.0 and SD = 0.2 in both groups
Allen et al. [24], 2014	Yes/no to colic symptoms (not defined)	Weeks 9–28	RR (95% CI) = 1.04 (0.77, 1.40)
Giglione et al. [32], 2016	Minutes of crying/d	Weeks 8–12	MD (95% CI) = −35.10 (−55.41, −13.51)
Hiraku et al. [35], 2023	Episodes/day with >30 min of crying	At 12 wk	MD (95% CI) = 0.05 (−0.02, 0.08)
Hoy-Schulz et al. [36], 2016	% of follow-up time with colic symptoms (not defined) per infant (questionnaire)	At 12 wk	Median (SD) = 0% (9.0) in controls 1.1% (3.9) in probiotic group
Maldonado et al. [42], 2019	Continuous and disconsolate crying episodes >3 h/d during the last 2 d	At 6 wk^1^	RR (95% CI) = 0.49 (0.23, 1.01)
Smilowitz et al. [49], 2017	Crying for > 3 h/d for ≥3 d/wk—questionnaire	Weeks 4–8	RR (95% CI) = 0.67 (0.12, 3.76)

Abbreviations: CI, confidence interval; MD, mean difference; RR, risk ratio.

1 Clear bias of reporting, because this timepoint is an outlier—(Figure 3 [42]).

Apart from colic-related outcomes, some of the included studies also reported AEs and growth parameters as measures of infant health.

Growth was included as a safety measure in 14 studies. Results were reported either as growth rates (e.g., g/d) or as measurements at baseline and at the end of follow-up (Supplemental Table 5). Alba et al. [22] reported a MD in weight of −0.41 kg (95% CI: −0.45, −0.37] and an increase in height of 2.40 cm (1.45, 3.35). This apparent reduction in weight gain and increase in height among probiotic groups was not observed in any other study, except for Hiraku et al. [35], who also reported an MD in height of 0.24 cm (0.14, 0.34).

Nine studies included a total number of participants who suffered any AE. The forest plot (Supplemental Figure 11) includes the studies reporting the number of infants with >1 AE. Two studies [33,47] only reported the total number of incidents and have not been included. There is no significant difference in AEs in any of the 9 studies.

## Discussion

### Main findings

#### Overall

This review includes 25 studies assessing the effects of ITB-probiotics on various clinical outcomes. Notably, there is substantial variability across all components of the PICO model (Table 1). Infant populations range from prenatal exposure to 4 mo of age at first dose, with some studies including relatively small sample sizes. Interventions vary from isolated ITB supplementation to more complex infant formulas containing ITB as an additive, with intervention durations ranging from just a few weeks to nearly a year.

High heterogeneity was observed in the meta-analyses related to RTI stools per day, diarrhea, and constipation. This heterogeneity was likely driven by differences in outcome definitions (as detailed in Table 3) and variation in follow-up periods. For stool frequency in particular, a subgroup difference based on follow-up duration (<16 wk compared with ≥16 wk) further supported the role of follow-up time as a source of heterogeneity.

Outcome heterogeneity was an inherent aspect of this review. However, all included studies were RCTs, which reduces variability in study design. Differences in outcome definitions and data sources (e.g., parent-reported compared with clinically assessed) should still be considered when interpreting the applicability of the meta-analyses. Although most pooled estimates had 95% CIs covering the null effect, 9 meta-analyses showed an RR < 1, suggesting a tendency toward risk reduction, and 2 (allergic rhinitis, and AEs ) showed an increased relative risk.

Five studies were assessed as having a high RoB, primarily due to issues with the randomization process or the absence of a placebo control [25,29,30,31,49]. As illustrated for “Eczema” in Supplemental Table 3, exclusion of these studies did not materially alter the pooled estimates, and for the remaining outcomes, these studies contributed only minimal statistical weight, except for “Respiratory infections,” for which the results are presented excluding them. The remaining studies were generally of high quality with a low RoB. In studies assessed as having “Some concerns,” the main source of bias was incomplete reporting of dropouts.

The Kukkonen et al. trial constitutes the largest study included in our review and therefore contributes substantially to the weight of several outcomes. Leave-one-out sensitivity analyses show that exclusion of this study does not materially change the overall estimates or conclusions (Supplemental Table 2). The trial is assessed as high quality with low risk of bias across all 5 RoB-2 domains.

#### Infections, fever, and antibiotic use

Although not statistically significant, the observed reduction in antibiotic use among infants receiving probiotics may indicate a clinically relevant trend. These results should be interpreted with caution; however, the direction of effect is supported by the fact that none of the studies included reported an RR > 1.00. Notably, the Kukkonen et al. trial found a statistically significant reduction in antibiotic use during the first 6 mo of life in the probiotic group, although this difference was attenuated at the 10-y follow-up.

The meta-analysis shows a borderline significant 26% risk reduction of RTI development in infants receiving ITB in alignment with previous findings regarding probiotic-enriched formulas [61].

It is possible that a reduction in RTIs could influence antibiotic prescriptions. Only 5 of the included studies reported both antibiotic use and RTI development [22,23,30,37,49], with 3 of these reporting a risk reduction for both outcomes [22,23,30]. This could also be influenced by prescribing practices, parental expectations, and broader immunomodulatory effects of probiotics, which may reduce the need for antibiotics independently of RTI occurrence.

The analyses indicated no clear preventive effect on overall infection prevalence or fever. However, the meta-analyses suggested a tendency toward reduced risks. A previous meta-analysis on the efficacy of probiotics in preventing urinary tract infections in children found no notable benefits, apart from a moderate effect when used as an adjunct to antibiotics [54]. Future studies are urged to distinguish between bacterial and viral infections, as well as the specific organs involved. The lack of data regarding the definition of fever underscores the need for clear, consistent criteria to improve data reliability and clinical applicability.

#### Atopy, allergy, and asthma

The meta-analysis showed a 22% statistically significant risk reduction regarding development of eczema/atopic dermatitis, a result of great clinical importance. It aligns with the results of previous studies and meta-analyses evaluating effects of a broader spectrum of infant probiotics on the development of eczema [10,11]. They observed a stronger effect of prenatal administration, which is also suggested by our findings (Supplemental Table 3).

Despite the established link between atopic dermatitis, food allergy (FA), asthma, and allergic rhinitis, “the atopic march” [55]—this review only observed a risk reduction for eczema. However, Kukkonen et al. indicated associations to both asthmatic bronchitis and FA development during the 13-y follow-up period. The potential link to FA was further supported by a significant result from Allen et al. [23,24], who also used a positive skin prick test as the outcome definition in contrast to self-reporting in many of the other studies. Definitions and prevalence rates for these conditions vary significantly across studies, leading to considerable heterogeneity.

The subgroup analysis comparing ITB to multistrain probiotics highlights that an ITB supplement alone achieves a similar clinical outcome. This emphasizes the clinical relevance of studying specific probiotic strains rather than treating all probiotics as a homogeneous group.

When comparing populations of only formula-fed to mixed-fed infants, a potential further risk reduction was observed in the mixed-fed group. This finding suggests that the HMOs present in breastmilk may be important to maximize the efficacy of ITB-probiotics. No other subgroup analyses demonstrated major differences (Supplemental Table 3).

It is also important to note that although only 1 study focused on exclusively breastfed infants, 13 studies examined populations receiving only formula (Table 1). Given the findings of the subgroup analysis, this may influence the applicability of the review’s results, depending on the cultural and clinical context.

#### Gastrointestinal health and tolerability

In studies evaluating stool frequency from week 16 onward, there was a slight increase in the number of stools per day in the ITB intervention group. Stool frequency changes with infant age, with higher frequencies in early infancy because of immature gut function and rapid transit. Stratifying studies at 16 wk may account for some developmental differences in gut motility, microbiota, and dietary transitions that may influence stool patterns [62]. Despite the observed increase in stool frequency, the meta-analysis suggests a preventive effect of ITB-probiotics on diarrhea, even though the outcome was primarily defined by stool frequency. In addition, a review of systematic reviews regarding the prevention of antibiotic-associated diarrhea in children concluded that probiotics are effective in both preventing and treating antibiotic-associated diarrhea [63]. Given this apparent contradiction, further research is needed to determine whether ITB-probiotics effectively prevent diarrhea in healthy infants.

#### Well-being and growth

According to Wessel's criteria, colic is defined as: “Infants affected by colic experience bouts of fussiness and crying that last ≥3 hours a day, for 3 or more days a week, for over 3 weeks” [64]. However, the definitions of colic were vague and varied in the studies included, and results were overall inconclusive. One study reported statistically significant reduced crying time in the probiotic group, aligning with existing literature on the impact of probiotic administration [58].

The studies assessing growth focused mainly on ruling out any growth challenges in the treatment groups, primarily as a safety measure. This may explain the short follow-up periods and insufficient data (e.g., reliance on medians, absence of SDs, or lack of participant numbers). A review from 2015 suggests a positive effect of probiotics on growth in malnourished children, whereas studies involving well-nourished children showed no effect [60].

#### Adverse events

The meta-analysis of AEs indicates a similar occurrence of AEs between the groups receiving ITB and the comparator group. Because the studies classified many different outcomes as AEs, a random distribution of relative risks (RRs) clustering around 1 was expected.

### Limitations

A key limitation of this review was the considerable heterogeneity among the included studies. Differences in study design, interventions, outcome definitions, and follow-up periods posed significant challenges for direct comparison and synthesis of findings. Also, the small number of studies on ITB-probiotics in healthy infants constrained the potential for meaningful subgroup analyses, despite most studies highlighting this need. Therefore, some outcome groups (e.g., “upper RTI” and “any RTI”) were combined.

The early-life gut microbiome is a dynamic and foundational ecosystem that seems to play a central role in shaping immune and metabolic development [59,65]. In this review, however, we chose to focus on clinical outcomes and not the actual gastrointestinal colonization, metabolomic, and biological processes after the studies interventions with ITB. Also, factors such as delivery mode and antibiotic use during pregnancy, which influence the infant's microbiota [66], were not assessed because of insufficient data and variations in exclusion criteria. Further studies on causality and microbiological pathways are needed to potentially explain the influence on clinical outcomes. However, several clinical trials and translational studies have demonstrated that supplementation with ITB, particularly *B. infantis,* results in a stable colonization of the infant gut [56,67,68].

### Strengths

This review provides a comprehensive assessment of all major clinical outcomes, after ITB-probiotics to infants, offering valuable new insights on potential effects. It addresses a current knowledge gap regarding how probiotics could be used in a clinical or preventative setting.

A thorough and systematic literature search was conducted across multiple databases, including PubMed, Embase, CENTRAL, and Scopus, ensuring the inclusion of all relevant studies. The search was also updated to capture the most current evidence. In addition, a risk-of-bias assessment was performed using the revised Cochrane tool (RoB 2), further strengthening the reliability of the conclusions.

In conclusion, this systematic review suggests that probiotics containing ITB may reduce risk of RTI and eczema in healthy infants, based on evidence from RCTs. With its specific focus on ITB-probiotics in infancy, the review provides a comprehensive synthesis of the evidence across multiple outcomes, offering an integrated perspective on their potential effects. The subgroup analyses of studies with eczema as an outcome did not reveal any effect modifiers or significant biases within the predefined groupings based on existing literature. However, the feeding pattern of the infants emerged as a potential factor, with ITB-probiotics appearing to have a greater effect when combined with breastfeeding.

For other outcomes—including antibiotic use, fever, asthmatic bronchitis, FA, allergic rhinitis, diarrhea, constipation, and AEs —meta-analyses showed no statistically significant effects. However, relative risk estimates for most of these outcomes tended to be <1, suggesting possible protective effects that warrant further investigation, though conclusions should remain cautious in the absence of statistical significance.

Future research should not only address the short-term clinical outcomes but also explore potential long-term effects of ITB-probiotics. Comprehensive RCTs with longer follow-up, larger sample sizes, and standardized clinically relevant outcome measures are necessary to understand the potential effects of ITB on remaining atopic diseases beyond eczema, as well as on gastrointestinal health and growth. If confirmed in future investigations, these findings could inform clinical guidelines and support public health strategies aimed at preventing disease and improving global health.

## Author contributions

The authors’ responsibilities were as follows – MAS, MTP, TBH, SLMR: designed the review; MAS, JS: conducted research and analyzed data; MAS, MTP, TBH, SLMR: wrote the article; MAS: had primary responsibility for final content; and all authors: read and approved the final manuscript.

## Data availability

Data described in the manuscript and supplementary material, including risk-of-bias assessments and subgroup analyses, are available from the corresponding author on reasonable request (e-mail: mathiv@rm.dk).

## Declaration of Generative AI and AI-assisted technologies in the writing process

During the preparation of this work the author(s) used Microsoft Copilot to provide suggestions to improve readability. After using this tool, the authors reviewed and edited the content as needed and take full responsibility for the content of the publication.

## Funding

The authors reported no funding received for this study.

## Conflict of interest

The authors report no conflict of interest.
