# Supplementary material for: Probiotics in Term Infants: Clinical Impact of Infant-Type Bifidobacteria: A Systematic Review and Meta-analyses
Source: J Nutr. 2025 Oct 9;155(12):4075–86. doi: 10.1016/j.tjnut.2025.10.006 (PMC12799449; doi:10.1016/j.tjnut.2025.10.006)
Supplement: Multimedia component 1 [file mmc1.docx]

**Probiotics in Term Infants:
Clinical Impact of Infant-Type Bifidobacteria
- A Systematic Review and Meta-Analyses**

Mathias A. Sjælland^1^

**Supplementary material:**

**Figure S1.** Search- screening- and inclusion process. Figure created in Covidence.


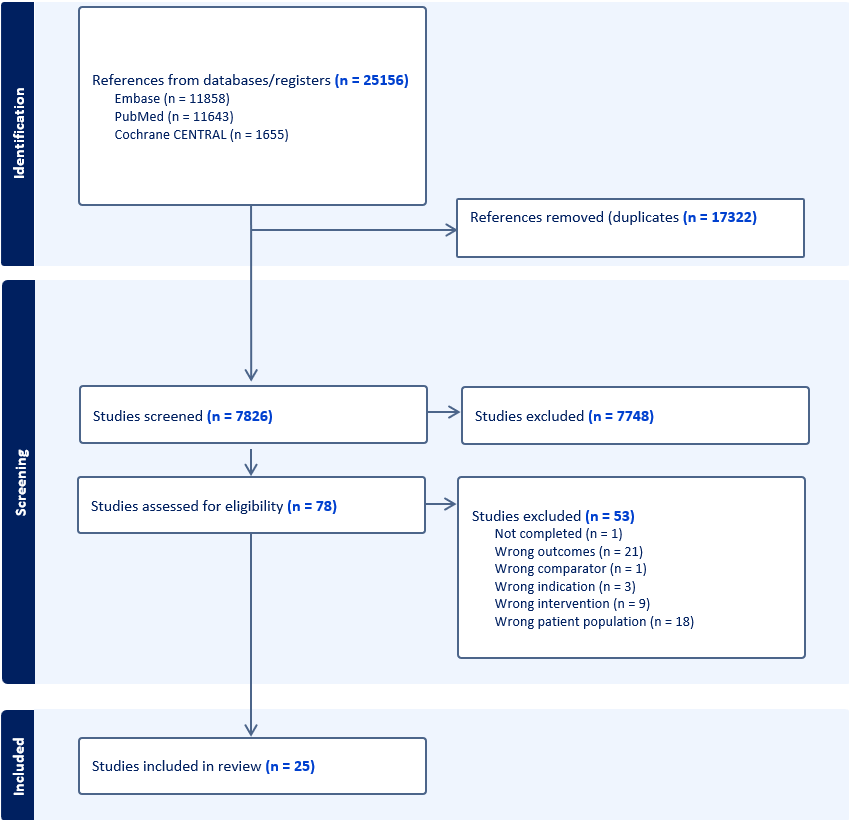


**Table S1.** The outcomes are divided into continuous and dichotomous data. When not explained further, dichotomization is based on “any” occurrence of the outcome of interest. Only outcomes with relevance for the purpose of this review are defined.

| Study | Dichotomous outcomes | Continuous outcomes |
| --- | --- | --- |
| Abrahamse-Berkeveld, 2016 | Antibiotics  Adverse event (AE) | Diaper rash severity  Vomiting severity  Diarrhea severity  Flatulence severity  Colic severity  Growth  Stool frequency  Stool consistency |
| Alba, 2024 | Antibiotics  Respiratory tract infections  Diarrhea*** | Growth |
| Allen, 2014 | Antibiotics  (Infections)  Upper respiratory tract infections  Eczema  Unscheduled doctor visit  Asthmatic bronchitis  Food allergy  Allergic rhinitis  Wheezing  Colic  Diarrhea  Constipation  AE | Weight change |
| Bellomo, 2024 | Antibiotics**  Upper respiratory tract infections  Lower respiratory tract infections  Eczema  Diarrhea**  Constipation**  Dyspeptic syndrome (not defined) | Weight gain |
| Capeding, 2023 | Fever  Upper respiratory tract infections  AE | Growth  IGSQ-score  Number of regurgitations/vomiting  Stool frequency  Stool consistency |
| Chouraqui,  2008 | Antibiotics  Hospitalizations  Diarrhea  AE | Growth |
| Dissanayake, 2019 | Eczema  Food allergy |  |
| Enomoto, 2014 | Eczema  Asthmatic bronchitis  Allergic rhinitis |  |
| Escribano, 2019 | Antibiotics  Respiratory infections  Diarrhea  Constipation  Vomiting | Diarrhea severity  Stool frequency  Stool consistency*  Sleeping behavior |
| Giglione, 2016 |  | Crying time  Growth  Vomiting  Stool frequency  Stool consistency |
| Harvey, 2014 | Infections  Eczema  Diarrhea | Vomiting severity**  Flatulence severity**  Episodes of colic **  Number of AE’s  Stool frequency**  Stool consistency ** |
| Hascoët, 2011 | Upper respiratory tract infection  AE | Growth |
| Hiraku, 2023 | Fever  Eczema  Diarrhea  Constipation  Vomiting  AE | Growth  Crying time  Stool frequency*  Stool consistency* |
| Hoy-Schulz, 2016 | Hospitalization | Diarrhea severity  Vomiting severity  Episodes of colic |
| Kukkonen, 2007 | Antibiotics  Upper respiratory tract infections >4 pr year  Respiratory infections  Eczema  Food allergy  Asthmatic bronchitis  Allergic rhinitis  Wheezing | Growth |
| Maldonado, 2019 | Eczema  Colic | Fever  Upper respiratory tract infections  Lower tract respiratory infections  Conjunctivitis  Otitis  Urinary tract infections  Diarrhea events  Growth  Stool frequency*  Stool consistency* |
| Manzano, 2017 | Infections  AE | Growth  Stool frequency  Stool consistency Fever episodes  Diarrhea episodes  Unscheduled doctor visits Crying episodes |
| Niers, 2009 | Eczema  Food allergy  Asthmatic bronchitis  Allergic rhinitis |  |
| Phavichitr, 2021 |  | Stool frequency*  Stool consistency* |
| Puccio, 2007 | Respiratory tract infections | Growth  Number of AE’s  Stool frequency  Stool consistency* |
| Rozé, 2012 | Antibiotics  Eczema | Growth  Stool frequency |
| Smilowitz, 2017 | Antibiotics  Respiratory tract infections  Urinary tract infections  Ear infections  Eczema  Astmatic bronchitis  Wheezing  Allergic reaction  Chronic diarrhea  Hospitalization  Unscheduled doctor visit  Colic | Febrile episodes  Extent of chronic diarrhea  Stool frequency*  Stool consistency* |
| Soh, 2009 | Antibiotics  Eczema  Food allergy |  |
| Wang, 2021 | Infections  Eczema  Vomiting  AE | Growth  Stool frequency  Stool consistency* |
| Xiao, 2019 | AE | Growth  Stool frequency |

*IGSQ = Infant Gastrointestinal Symptom Questionnaire
*The data was presented with an insufficient level of detail and was not obtained after contacting the authors.
** The data were reported with insufficient specificity to allow for the reporting of comparable results.
*** Reported as “Gastrointestinal infections” but defined as “loose or watery stools at least three times per day with or without fever or vomiting”.*

**Table S2.** Leave-one-out meta-analyses of outcomes with <5 studies in which a single trial contributed more than 40 % of the statistical weight.

| **Outcome** | **RR (95% CI)** | **RR (95% CI) after leave-one-out analysis** |
| --- | --- | --- |
| **Any antibiotic use** | 0.92 [0.81, 1.04] | 0.80 [0.63, 1.02], excl. Kukkonen (2007) |
| **Any respiratory infection** | 0.74 [0.54, 1.00] | 0.60 [0.45, 0.81], excl. Kukkonen (2007) |
| **Eczema** | 0.78 [0.67, 0.89] | 0.75 [0.62, 0.91], excl. Kukkonen (2007) |

RR = Risk Ratio, CI = Confidence Interval, excl. = excluding.

**Figure S2.** Forest plot of “Any fever”

**Figure S3**. Forest plot of “Any infections”

**Table S3.** Subgroup analyses of “any eczema/atopic dermatitis” development. Subgroup distribution in Table 1.

| **Subgroup 1** | **Subgroup 2** | **RR1 [95 % CI]** | **RR2 [95 % CI]** |
| --- | --- | --- | --- |
| Synbiotics [23,26,29,31,56] | Probiotics [22,24,25,30,32,35,28,34] | 0.81 [0.68, 0.97] | 0.73 [0.58, 0.91] |
| Only infant-type bifidobacteria (ITB) [22,23,24,25,26,34,35,56] | ITB + other strain(s) [28,29,30,31,32] | 0.78 [0.55, 1.11] | 0.78 [0.67, 0.91] |
| Infants predisposed to eczema* [28,29,30,32] | “Normal” population [22,23,24,25,26,31,34,35,56] | 0.79 [0.67, 0.92] | 0.70 [0.48, 1.03] |
| Some prenatal administration [29,30,31,34] | Only postnatal [22,23,24,25,26,28,32,35,56] | 0.76 [0.64, 0.89] | 0.86[0.65, 1.14] |
| Only formula fed [23,25,26,31,32] | Mixed feeding [22,24,28,29,30,34,35,36] | **0.89 [0.64, 1.24]** | **0.75 [0.64, 0.88]** |
| Outcome measured at ≥ 12 mo [22,25,28,29,30,32,34,35,56] | <12 mo [23,24,26,31] | 0.79 [0.68, 0.92] | 0.63 [0.32, 1.25] |
| Risk of bias (ROB) = low/some concerns [23,24,25,26,28,29,30,31,32,56] | High ROB [22,34,35] | 0.77 [0.66, 0.90] | 0.70 [0.39, 1.26] |

mo = months, RR = Risk Ratio, CI = Confidence Interval
Values in bold denote statistically significant results (p < 0.05)
* Family history of allergic disease was an inclusion criterion

**Figure S4.** Forest plot of “Any asthmatic bronchitis”

**Figure S5.** Forest plot of “Any food allergy”


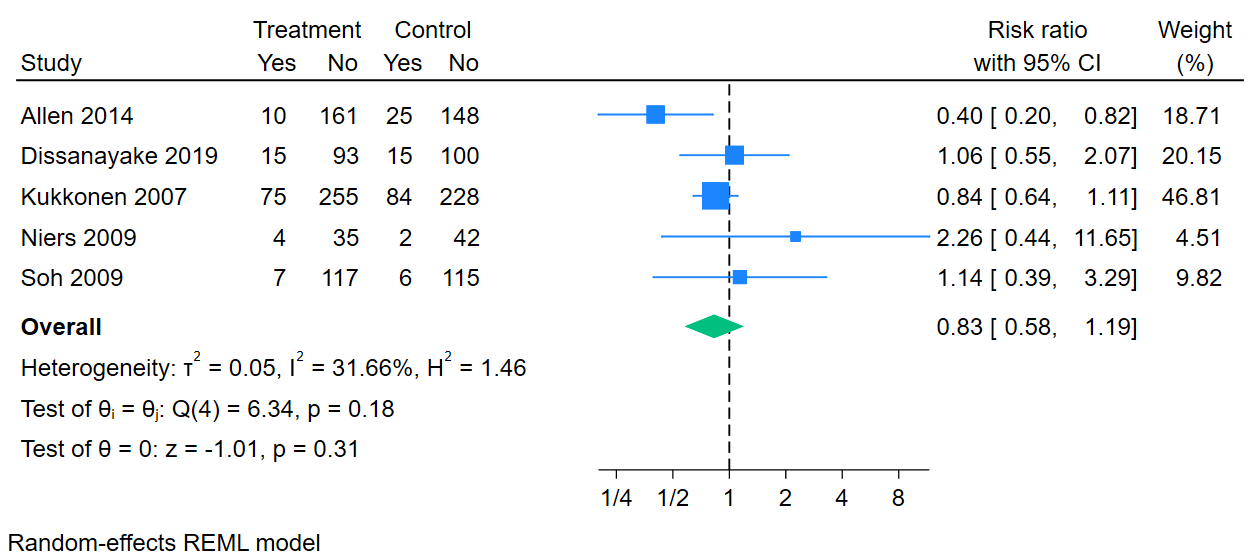


**Figure S6.** Forest plot of “Any allergic rhinitis”


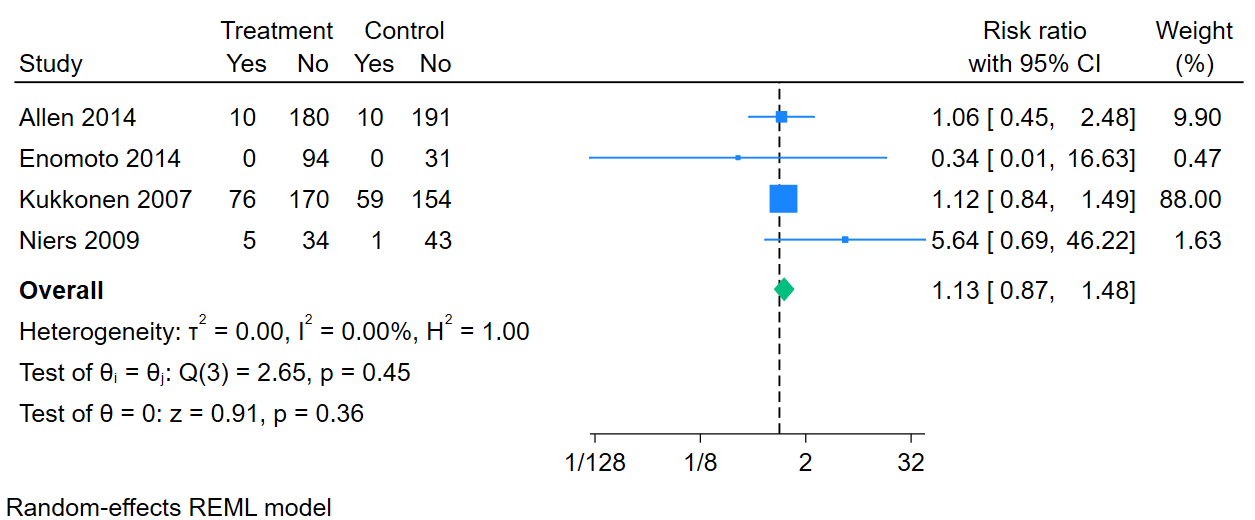


**Figure S7.** Forest plot of “Stool frequency (stools/day)”

**Figure S8.** Forest plot of studies evaluating stool frequency after week 16 (4 months).

**Figure S9.** Forest plot of “Any diarrhea”

**Figure S10.** Forest plot of “Any constipation”

**Figure S11.** Forest plot of “Any adverse events”

**Table S4**. Elaboration of outcomes and results concerning vomiting or vomiting/regurgitations (but not regurgitations alone).

| **Study** | **Outcome measure** | **Follow-up period** | **Result** |
| --- | --- | --- | --- |
| Abrahamse-Berkeveld et al., 2016 | Severity scores: 0 = absent; 1 = mild; 2 = moderate; 3 = severe | 13 weeks | Very low prevalence.  Mean (synbiotic) = 0.1 (SD=0.2) Mean control = 0.0 (SD=0.2)  (From week 8-13) |
| Capeding et al., 2023 | Regurgitations/vomiting. Groups of:  0 times, 1 time, 2-3 times, 4-6 times, 7 or more times. | 14 weeks | “At Visit 14 weeks, frequency of vomiting  was lower in both Hi-EG (high dose) and Lo-EG (low dose) compared with control group (value of  p=0.044 and 0.006, respectively)” |
| Escribano et al., 2019 | % of children with more than one vomit/day | 12 weeks | 6.3 % in control group.  6.0 % in probiotic group. |
| Giglione et al., 2016 | Episodes of vomit(/day) | 12 weeks | 0.11 (SD=0.06) in probiotic group.  0.2 (SD=0.07) in control group.  “Episodes of vomits decreased significantly with time in the probiotic group but not in the placebo group (p < 0.03)” |
| Hiraku et al., 2023 | Episodes of vomit(/day) and % of infants vomiting | 12 weeks | 0.03 (SEM=0.01) in control group.  0.11 (SEM=0.06) in probiotic group.  p=0.087   9.4 % in control group  21.4 % in probiotic group  p=0.114 |
| Hoy-Schulz et al., 2016 | Median percent of follow-up time with symptoms per infant | 12 weeks | 0 in both groups. |
| Wang et al., 2021 | % with one occurrence of vomiting | 17 weeks | 38.5 % in placebo group  39.3 % in probiotic group. |

SD = Standard deviation, SEM = Standard Error of the Mean

**Table S5**. Growth parameters**.** Results reported as the mean difference (MD) of the rate (g/d e.g.) or an absolute number (between end results or a change from baseline(Δ)) in accordance with article data. Placebo-intervention.

| **Study** | **Time period** | **Weight** | **Height** | **Head circumference** |
| --- | --- | --- | --- | --- |
| Abrahamse-Berkeveld et al., 2016 | 3 months | 1.0 [0.81, 1.19] g/d | 0.04 [0.035, 0.045] cm/week | 0.02 [0.017, 0.023] cm/week |
| Alba et al., 2024 | 3 months | **-0.41 [-0.45, -0.37] kg** | **2.40 [1.45, 3.35] cm** | 0.30 [-0.15, 0.75] cm |
| Allen et al., 2014 | 6 months | -0.07 [-0.91, 0.76] kg |  |  |
| Capeding et al., 2023 | 3 months | -1.00 [-3.20, 1.20] g/d * | ** | ** |
| Chouraqui et al., 2008 | 12 months | -1.04 [-2.94, 0.90] g/d | 0.06 [-0.71, 0.83] (mm/mo) | -0.95 [-2.10, 0,20] mm/mo |
| Giglione et al., 2018 | 3.5 months | 13.4 [-5.7, 32.5] g | 0.00 [-0.10, 0.10] cm | -0.10 [-0.13, -0.07] cm |
| Hascoét et al., 2011 | 4 months | 0.30 [-2.40, 3.00] g/d | 0.02 [-0.07, 0.11] mm/d | -0.04 [-0.11, 0.03] mm/d |
| Hiraku et al., 2023 | 3 months | 29.45 [-7.31, 66.21] g | **0.24 [0.14, 0.34] cm** | -0.07 [-0.15, 0.01] cm |
| Kukkonen et al., 2007 | 24 months | 0.00 [-0.19, 0.19] kg | -0.20 [-0.61, 0.21] cm | -0.10 [-0.31, 0.11] cm |
| Maldonado et al., 2019 | 12 months | -0.14 [-0.59, 0.31] kg | 0.16 [-1.02, 1.34] cm | -0.42 [-0.95, 0.11] cm |
| Manzano et al., 2017 | 2 months | -0.06 [-0.23, 0.11] kg | -0.21 [-0.67, 0.25] cm | -0.02 [-0.27, 0.23] cm |
| Puccio et al., 2007 | 4 months | ** | 0.00 [-1.45, 1.45] mm/mo | 0.50 [-0.44, 1.44] mm/mo |
| Rozé et al., 2012 | 6 months | 245.90 [-137.28, 629.01] g Δ | 0.35 [-0.97, 1.67] cm | 0.24 [-0.38, 0.86] cm |
| Wang et al., 2021 | 4 months | -0.40 [-0.59, -0.21] g/d | -0.01 [-0.02, 0.00] mm/d | 0.01 [0.00, 0.01] mm/d |
| Xiao et al., 2019 | 1 month | 32.10 [-66.15, 130.35] g Δ | 0.3 [-0.13, 0.73] cm Δ | -0.1 [-0.33, 0.13] cm Δ |

Values in bold denote statistically significant results (p < 0.05)
Abrahamse-Berkeveld et al., 2016: The small SD values may reflect standard errors; authors were contacted for clarification but did not respond
*Approximated from median and interquartile ranges using the method by Wan et al. (2014).
** Data only presented graphically in the article, no extractable numerical values available. No data were extracted from figures; all data were obtained directly from the original studies
